# Biomechanical Evaluation of Different Surgical Approaches for the Treatment of Adjacent Segment Diseases After Primary Anterior Cervical Discectomy and Fusion: A Finite Element Analysis

**DOI:** 10.3389/fbioe.2021.718996

**Published:** 2021-08-31

**Authors:** Wencan Ke, Chao Chen, Bingjin Wang, Wenbin Hua, Saideng Lu, Yu Song, Rongjin Luo, Zhiwei Liao, Gaocai Li, Liang Ma, Yunsong Shi, Kun Wang, Shuai Li, Xinghuo Wu, Yukun Zhang, Cao Yang

**Affiliations:** Department of Orthopaedics, Union Hospital, Tongji Medical College, Huazhong University of Science and Technology, Wuhan, China

**Keywords:** adjacent segment degeneration, finite element analysis, revision surgery, anterior cervical discectomy and fusion, laminoplasty

## Abstract

Symptomatic adjacent segment disease (ASD) is a common challenge after anterior cervical discectomy and fusion (ACDF). The objective of this study was to compare the biomechanical effects of a second ACDF and laminoplasty for the treatment of ASD after primary ACDF. We developed a finite element (FE) model of the C2-T1 based on computed tomography images. The FE models of revision surgeries of ACDF and laminoplasty were simulated to treat one-level and two-level ASD after primary ACDF. The range of motion (ROM) and intradiscal pressure (IDP) of the adjacent segments, and stress in the cord were analyzed to investigate the biomechanical effects of the second ACDF and laminoplasty. The results indicated that revision surgery of one-level ACDF increased the ROM and IDP at the C2–C3 segment, whereas two-level ACDF significantly increased the ROM and IDP at the C2–C3 and C7-T1 segments. Furthermore, no significant changes in the ROM and IDP of the laminoplasty models were observed. The stress in the cord of the re-laminoplasty model decreased to some extent, which was higher than that of the re-ACDF model. In conclusion, both ACDF and laminoplasty can relieve the high level of stress in the spinal cord caused by ASD after primary ACDF, whereas ACDF can achieve better decompression effect. Revision surgery of the superior ACDF or the superior and inferior ACDF after the primary ACDF increased the ROM and IDP at the adjacent segments, which may be the reason for the high incidence of recurrent ASD after second ACDF.

## Introduction

Anterior cervical discectomy and fusion (ACDF) is generally accepted as the standard surgical treatment for cervical degenerative diseases (Oglesby et al., 2013; Kelly et al., 2018). ACDF is recognized as a comparatively safe procedure associated with few complications. However, adjacent segment disease (ASD), defined as new symptoms at nerve roots or myelopathy and new radiographic evidence of degenerative changes at adjacent segments of previously fused segments, is one of the major problems after ACDF (Hilibrand and Robbins, 2004). In a retrospective study of 177 patients who underwent ACDF, radiographic and clinical ASD were found in 92.1 and 19.2% of patients, respectively; approximately 7% of the patients required revision surgery (Chung et al., 2014). Another study reported an incidence of 2.4% per year of revision surgery at adjacent segments after primary surgery, and the authors estimated that 22% of patients would require second surgery due to symptomatic ASD within a decade (Lee et al., 2015).

ASD can occur in the superior, inferior, or both adjacent levels, depending on the levels affected. Considering the clinical situation and secondary preoperative imaging findings, ASD can be treated by second ACDF, laminoplasty, and posterior fusion (Wang F. et al., 2017; Cao et al., 2020). ACDF decompresses the nerve roots and myelopathy by removing the herniated disc and posterior osteophytes, followed by restoration of the disc height and cervical lordosis by cages and bone graft (Schroeder et al., 2016; Muzević et al., 2018). Cervical laminoplasty was considered to be an effective method for the treatment of cervical degenerative stenosis as it expands the stenosed spinal canal (Yeh et al., 2014; Kurokawa and Kim, 2015). Posterior decompression and fusion can decompress the spinal cord and achieve immediate stabilization, thereby, preventing the occurrence of kyphotic (Du et al., 2014). Therefore, the appropriate surgical approaches for the treatment of ASD after ACDF still need to be studied.

The finite element (FE) analysis is an important method to study the spinal biomechanics (Nikkhoo et al., 2019; Cai et al., 2020b; Mesbah and Barkaoui, 2020). The range of motion (ROM), intradiscal pressure (IDP), facet joint stress, and stress in the cord can be calculated and analyzed to evaluate the biomechanical effects of different spine surgeries (Mesbah et al., 2020; Nikkhoo et al., 2020; Srinivasan et al., 2021). FE analysis can also be used to assess the risk of complications of spinal surgery, such as degeneration and internal implants fractures. However, the biomechanical evaluation of different surgical approaches for the treatment of ASD after ACDF has not been reported. In the present study, FE models with superior and two-level ASD after C4-C6 ACDF were conducted. The aim of this study was to compare the biomechanical effects of a second ACDF and laminoplasty for the treatment of ASD after primary ACDF.

## Materials and Methods

### Construction of Intact Cervical Model (C2–T1)

**FIGURE 1:**
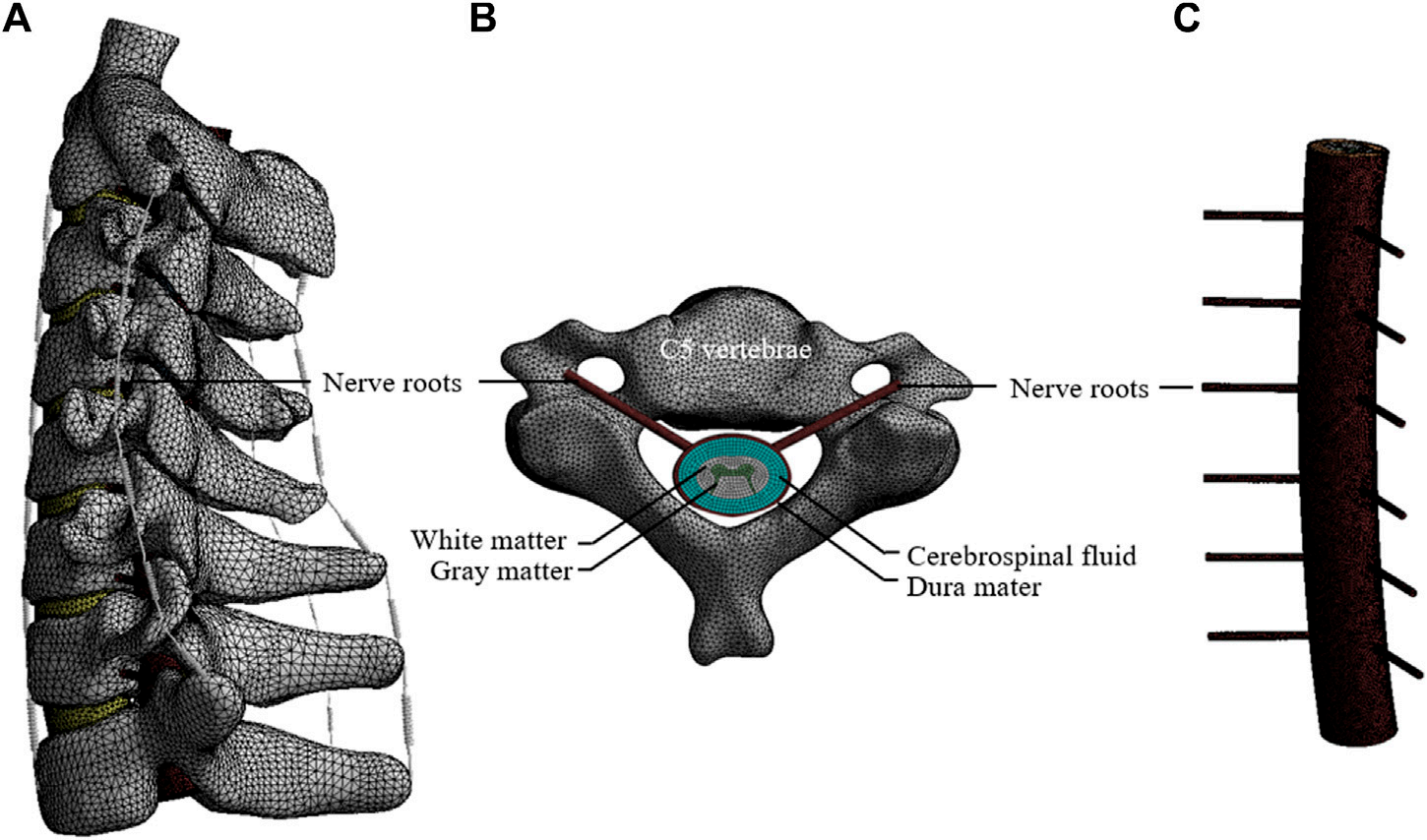
The FE model of the C2–T1. **(A)** The FE model of the cervical spine and spinal cord. **(B)** Axial view of the spinal cord at the C5 vertebrae. **(C)** Lateral view of the spinal cord model.

In this study, a three-dimensional FE model of C2–T1 segments was developed based on the computed tomography (CT) images of a healthy volunteer (male, 25 years old, 64 kg, and 176 cm). This study was approved by the ethics committee of Tongji Medical College, Huazhong University of Science and Technology. Written informed consent was obtained from the volunteer. The CT images of the participant were obtained at intervals of 0.625 mm (Dual Source CT; Siemens, Munich, Germany). Mimics Research 20.0 software (Materialize, Leuven, Belgium) was used to reconstruct the geometric structure of the vertebrae. Hypermesh (Altair Engineering, Troy, Michigan, United States) was used to mesh and build the FE models of C2-T1 vertebrae. Afterwards, the FE models were analyses by ANSYS (ANSYS Ltd., Canonsburg, Pennsylvania, United States). This C2–T1 FE model could be divided into cancellous bone, cortical bone, intervertebral disc (IVD), facet joints, and ligaments (Figure 1A). The cortical bone was constructed as a shell with the thickness of 0.4 mm (Mo et al., 2017). The IVD consisted of annulus fibrosus and nucleus pulposus with the volume ratio to be 6:4. The IVD was considered as an elastic material referring to the previous studies (Wu et al., 2019; Li et al., 2021). The endplates were constructed as a shell with the thickness of 0.5 mm. The facet joints were assumed with 0.5-mm thick cartilage with nonlinear, surface-to-surface, frictionless sliding contact (Li et al., 2017). The ligaments consisted of anterior longitudinal ligament, posterior longitudinal ligament, ligamentum flavum, interspinous ligament, supraspinous ligament, capsular ligament, and intertransverse ligament. These ligaments were established using nonlinear tension-only Spring element (Guo et al., 2021; Lin et al., 2021). The material properties of the model are listed in Table 2 (Cai et al., 2020a; Hua et al., 2020).

In addition, the spinal cord was reconstructed according to the geometry of the cervical column and human spinal cord. The spinal cord model included white matter, gray matter, dura mater, nerve roots, and cerebrospinal fluid (CSF) layers (Figures 1B,C). The dural sheath was placed approximately 2.5 mm from the cord, since the CSF layer in the human cervical spine was reported to be 1.5–4.0 mm in a previous literature (Holsheimer et al., 1994). The white and gray matter were assumed as Hyperelastic element based on study (Ichihara et al., 2001). The dura mater and nerve roots were constructed with elastic element according to study (Persson et al., 2010). CSF was assumed as Newtonian fluid according to the viscosity of CSF (Brydon et al., 1995). A one-way Fluid-Solid Interaction analysis method was used to couple the interaction between the fluid and solid material. Material properties of the spinal cord model are listed in Table 1.

**TABLE 1:** Material properties of the spinal structures.

Component/materials	Young’s modulus E (MPa)	Poisson’s ratio	Element type
Cortical bone	12000	0.29	Shell93
Cancellous bone	450	0.29	Solid187
Posterior element	3500	0.29	Solid187
Facet cartilage	10.4	0.4	Solid187
Endplate	500	0.4	Shell93
Nucleus pulposus	1	0.49	Solid187
Annulus fibrosus	3.4	0.4	Solid187
Anterior longitudinal Ligament	30	0.3	Spring (tension only)
Posterior longitudinal Ligament	20	0.3	Spring (tension only)
Ligamentum flavum	1.5	0.3	Spring (tension only)
Capsular Ligament	20	0.3	Spring (tension only)
Interspinous Ligament	1.5	0.3	Spring (tension only)
Supraspinous Ligament	1.5	0.3	Spring (tension only)
Intertransverse Ligament	20	0.3	Spring (tension only)
PEEK	3000	0.3	Solid187
Bone graft	450	0.29	Solid187
Titanium alloy	110,000	0.3	Solid187
Degenerative annulus fibrosus	4	0.45	Solid187
Degenerative nucleus pulposus	4	0.49	Solid187
Osteophytes	450	0.23	Solid187

### One-Level and Two-Level ASD Models After C4-C6 ACDF

**FIGURE 2:**
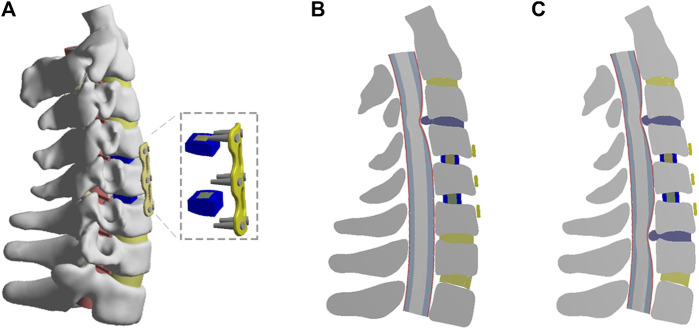
The FE models of C4-C6 ACDF and one-level or two-level ASD after primary ACDF. **(A)** The FE model of C4-C6 ACDF. **(B)** Cross-sectional views of the FE model of one-level ASD. **(C)** Cross-sectional views of the FE model of two-level ASD.

A recent study demonstrated that patients treated with one- or two-segment anterior cervical arthrodesis were more likely to develop ASD than those treated with three or more segments (Lee et al., 2015). Therefore, the ASD models after primary surgery were based on the C4-C6 ACDF model (Figure 2A). The C4-C6 ACDF model was constructed according to a previous study (Hua et al., 2020). In brief, annulus fibrosus and nucleus pulposus were partly resected and the polyetheretherketone (PEEK) cages with bone graft were placed in the intervertebral space. Then, solid fusion was achieved with anterior titanium alloy plates and titanium alloy screws. The one-level and two-level ASD models after C4-C6 ACDF were shown in Figures 2B,C. A moderate degeneration in the adjacent segment was modified to simulate ASD according to study (Cai et al., 2020a). The disc height was reduced by 50% relative to the height of the normal model. An osteophyte, one quarter of the size of the herniated disc, was constructed to simulate intervertebral disc calcification. An occupying ratio of 40% was assumed to simulate the spinal cord compression by ASD. The occupying ratio was defined as the ratio of the thickness of herniated disc to the anterior–posterior diameter of the spinal canal. The material properties of PEEK cages, bone graft, titanium alloy and degenerative intervertebral disc were also listed in Table 2.

**TABLE 2:** Material properties of the spinal cord.

Materials	Material model	Material parameters
White matter	Hyperelastic (Ogden)	μ = 4.0 kPa, α= 12.5
Gray matter	Hyperelastic (Ogden)	μ = 4.1 kPa, α= 14.7
Dura mater and nerve roots	Elastic	E = 80 MPa, ν= 0.49
Cerebrospinal fluid	Newtonian fluid	Viscosity = 0.001 Pa s

### Anterior Surgical Models for One-Level or Two-Level ASD After C4-C6 ACDF

**FIGURE 3:**
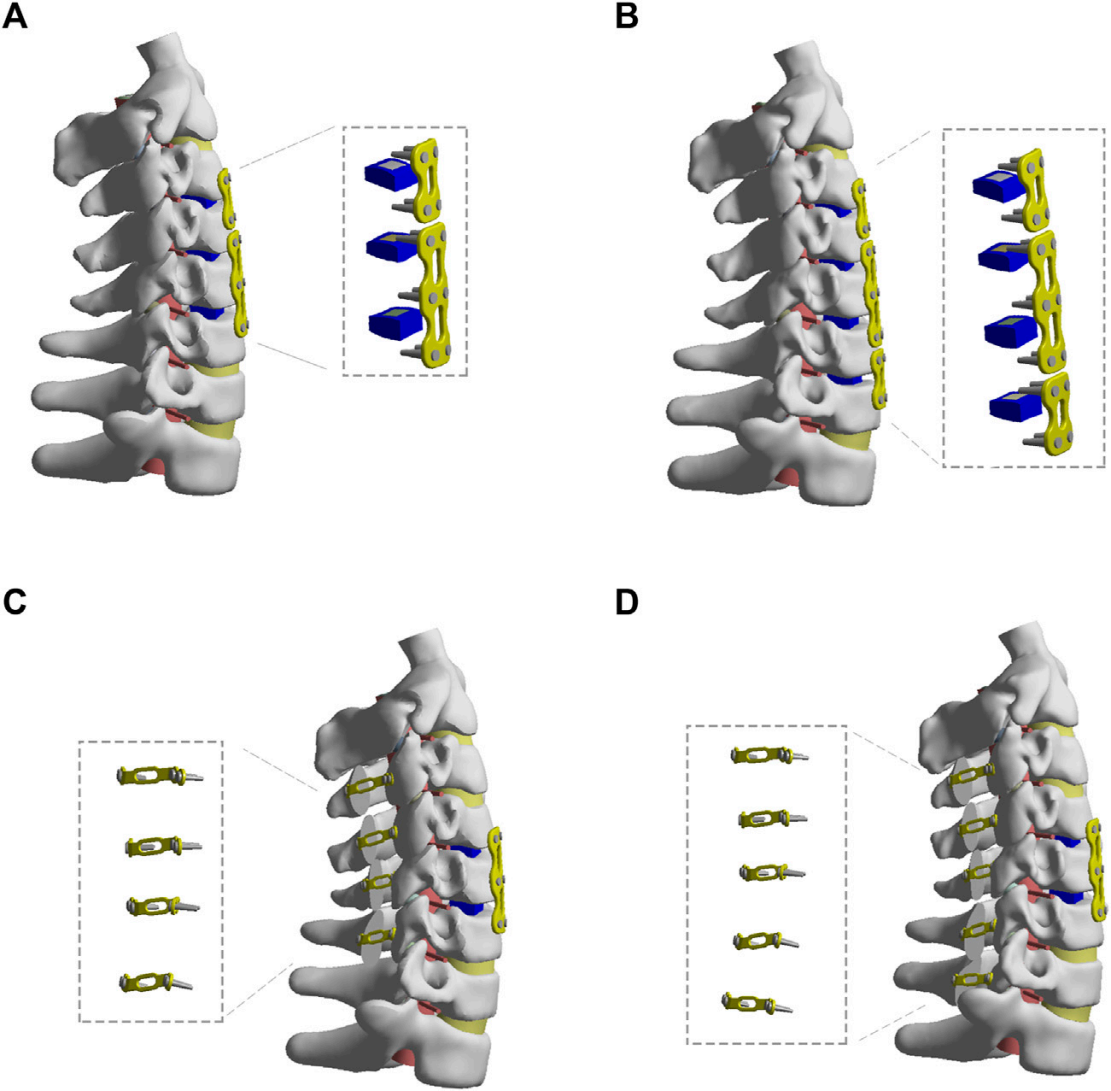
The FE models of different revision surgeries after C4-C6 ACDF. **(A)** The revision surgery of C2-C3 ACDF. **(B)** The revision surgery of C3-C4 and C6-C7 ACDF. **(C)** The revision surgery of C3-C6 laminoplasty. **(D)** The revision surgery of C3-C7 laminoplasty.

As shown in Figure 3A, for the treatment of one-level ASD after C4-C6 ACDF, an additional ACDF (re-ACDF) at C3-C4 level was constructed. As shown in Figure 3B, the second ACDF at C3-C4 and C6-C7 levels was simulated to treat two-level ASD after C4-C6 ACDF. The steps of ACDF are described above.

### Posterior Surgical Models for One-Level or Two-Level ASD After C4-C6 ACDF

As shown in Figures 3C,D, C3-C6 or C3-C7 laminoplasty (re-laminoplasty) was simulated to treat one-level or two-level ASD after C4-C6 ACDF, respectively. The laminoplasty models were developed based on conventional surgical protocols (Hirabayashi et al., 2010). Firstly, a longitudinal groove of 3 mm width was constructed between the lamina and lateral mass at hinge side of the lamina. Then, an opening width of 12 mm was made at the open side. The lamina was fixed using titanium alloy plates and screws.

### Boundary and Loading Conditions

All models were fixed at the inferior surface of the T1 vertebrae. A follower load of 73.6 N combined with a moment of 1.0 Nm was applied over the superior surface of C2 to simulate the spinal motions of flexion, extension, lateral bending, and axial rotation (Mo et al., 2017; Zhao et al., 2018). The ROM, IDP, and maximum von-Mises stress in the cord were analyzed to investigate the biomechanical effects of the second ACDF and laminoplasty for the treatment of ASD after primary ACDF.

## Results

### Model Validation

**FIGURE 4:**
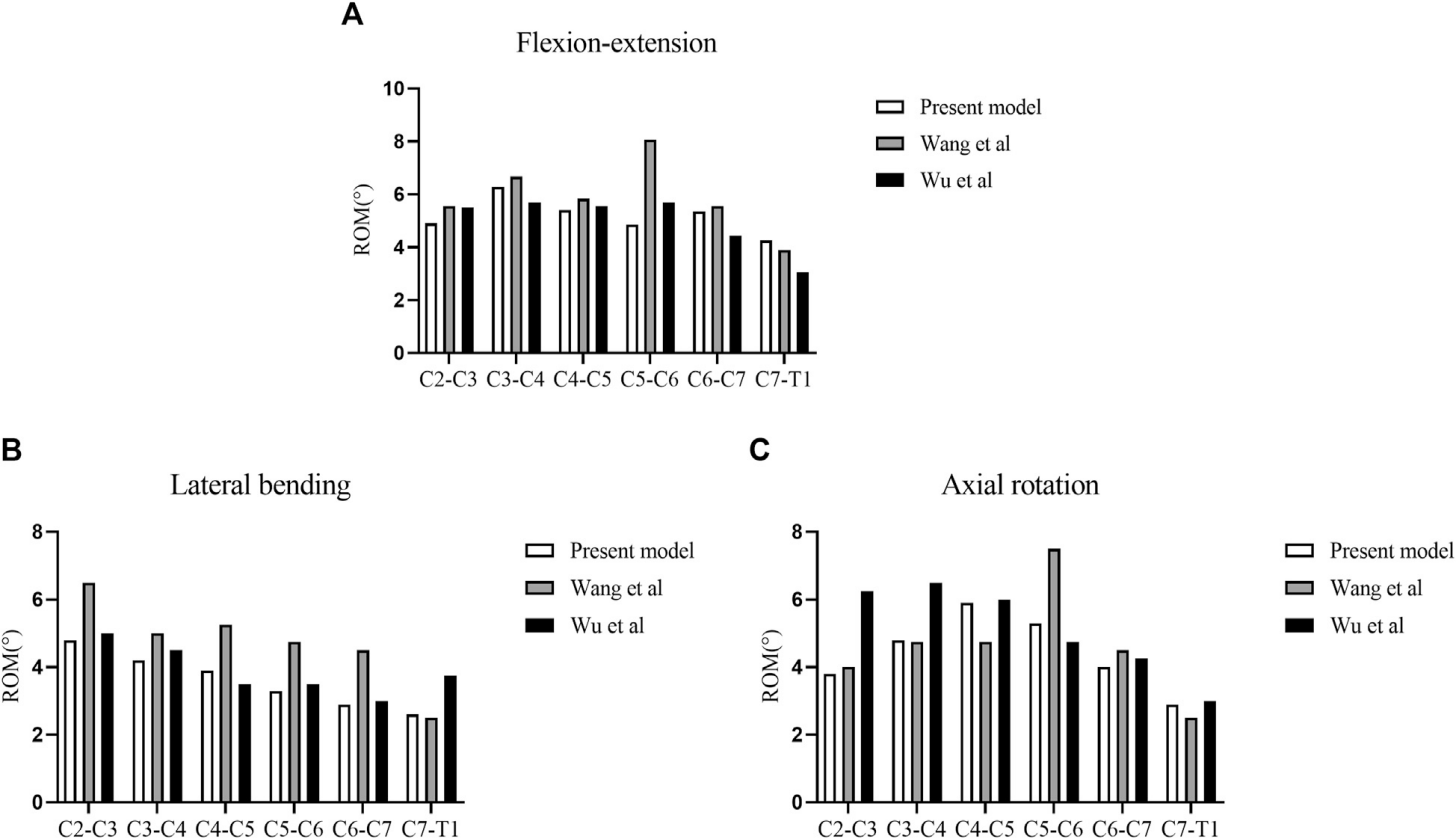
Validation of the intact cervical model. **(A)** ROM in flexion-extension. **(B)** ROM in lateral bending. **(C)** ROM in axial rotation.

The FE model of the intact cervical spine used in this study was validated by comparison with previous biomechanical models (Wang K. et al., 2017; Wu et al., 2018). The ROM and IDP of each segment were consistent with those of previous studies (Figure 4; Supplementary Figure S1).

### Analyses of the Biomechanical Effects of Different Surgical Approaches for the Treatment of One-Level ASD After Primary ACDF

**FIGURE 5:**
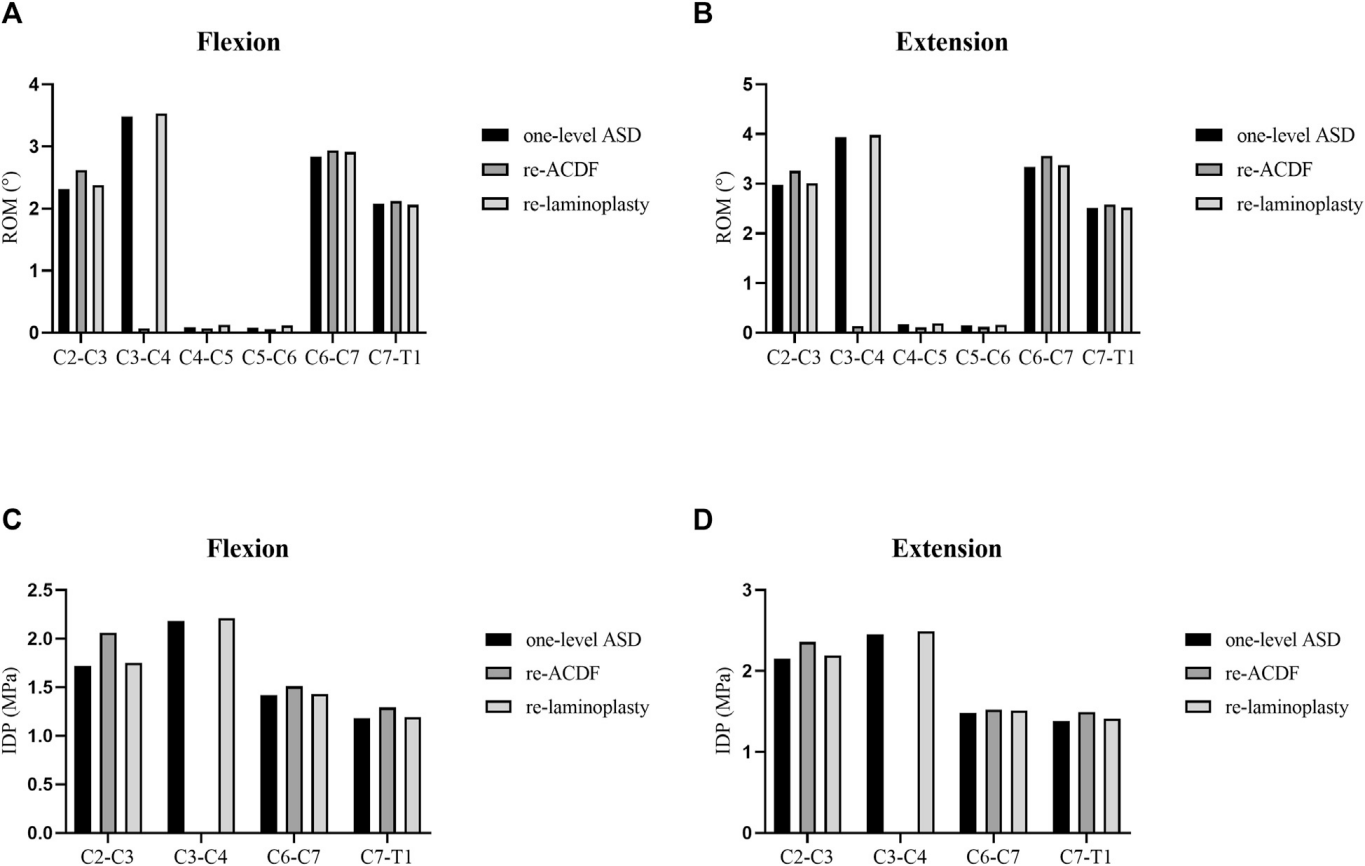
ROM and IDP of different revision surgeries for the treatment of one-level ASD. **(A)** ROM in flexion. **(B)** ROM in extension. **(C)** IDP in flexion. **(D)** IDP in extension.

The segmental ROM and IDP of the FE models of different surgical approaches for the treatment of one-level ASD after primary ACDF were shown in Figure 5. The ROM at the C2–C3 segment of re-ACDF model increased than that of the one-level ASD model (Figures 5A,B). Similarly, the IDP at the C2–C3 segment of re-ACDF model was larger than that of the one-level ASD model (Figures 5C,D). Furthermore, no significant changes in the ROM and IDP of re-laminoplasty model were observed compared to the one-level ASD model (Figure 5).

### Analyses of the Biomechanical Effects of Different Surgical Approaches for the Treatment of Two-Level ASD After Primary ACDF

**FIGURE 6:**
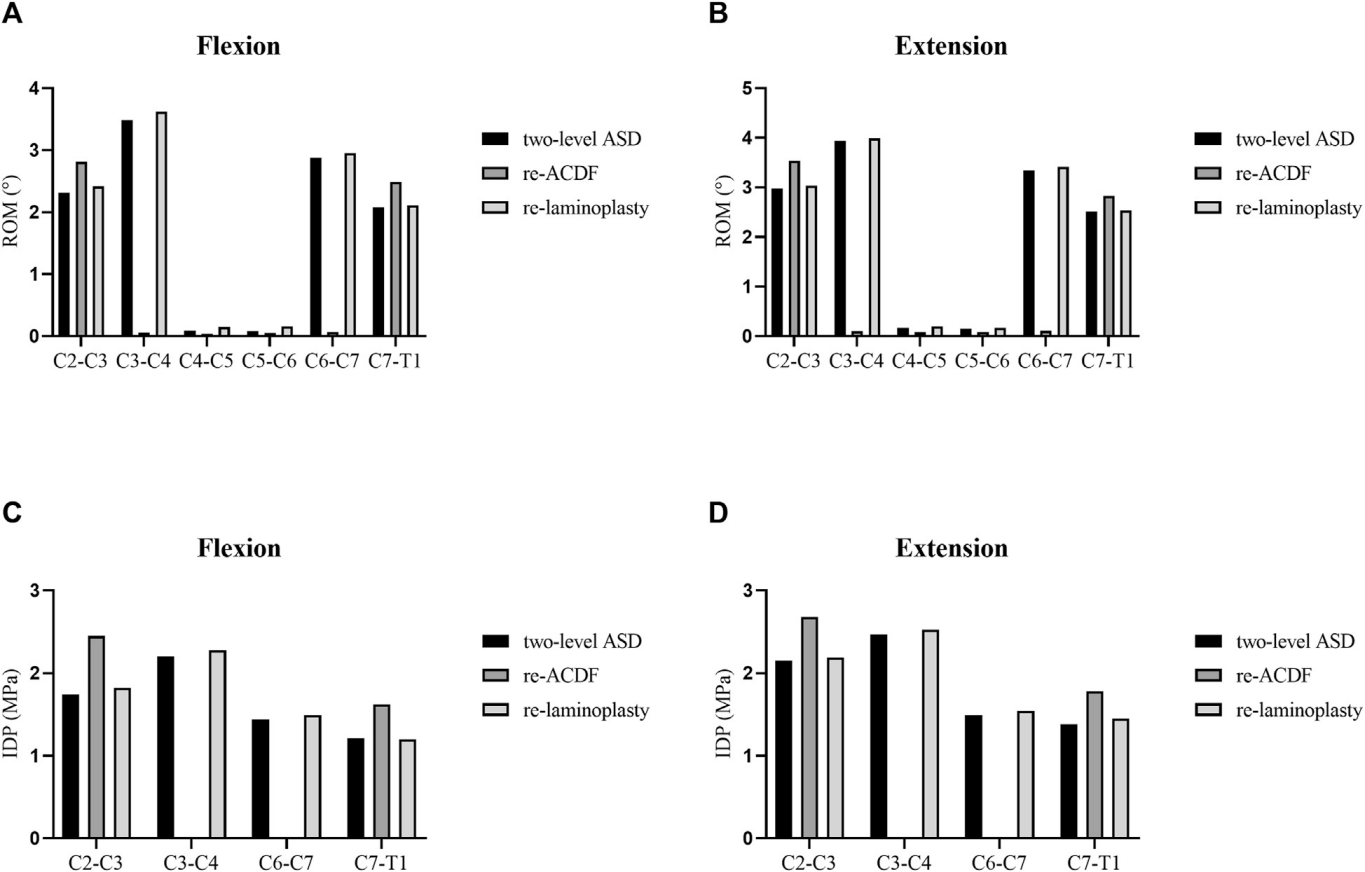
ROM and IDP of different revision surgeries for the treatment of two-level ASD. **(A)** ROM in flexion. **(B)** ROM in extension. **(C)** IDP in flexion. **(D)** IDP in extension.

The segmental ROM and IDP of the FE models of different surgical approaches for the treatment of two-level ASD after primary ACDF were shown in Figure 6. The ROM at the C2–C3 and C7-T1 segments of re-ACDF model increased significantly than that of the two-level ASD model (Figures 6A,B). Similarly, the IDP at the C2–C3 and C7-T1 segments of re-ACDF model was significantly larger than that of the two-level ASD model (Figures 6C,D). The ROM and IDP of re-laminoplasty model increased slightly but the difference was not statistically significant (Figure 6).

### Analyses of the Stress in the Spinal Cord of Different Surgical Approaches for the Treatment of One-Level or Two-Level ASD After Primary ACDF

**FIGURE 7:**
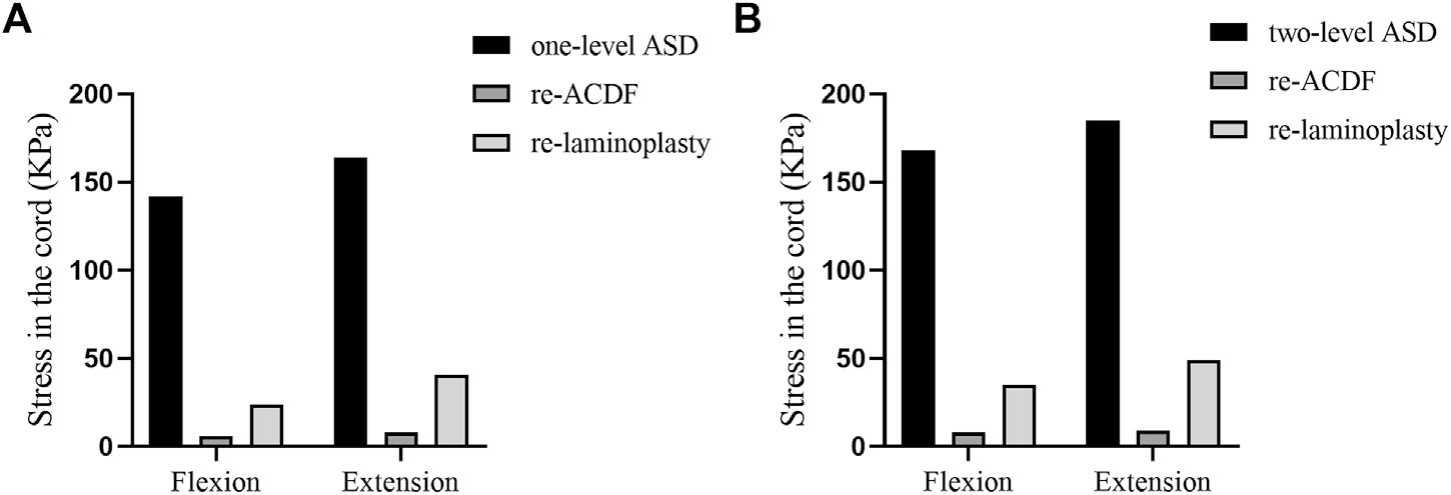
Maximum von-Mises stress in the cord of re-ACDF and re-laminoplasty models. **(A)** Stress in the cord of different revision surgeries for the treatment of one-level ASD. **(B)** Stress in the cord of different revision surgeries for the treatment of two-level ASD.

**FIGURE 8:**
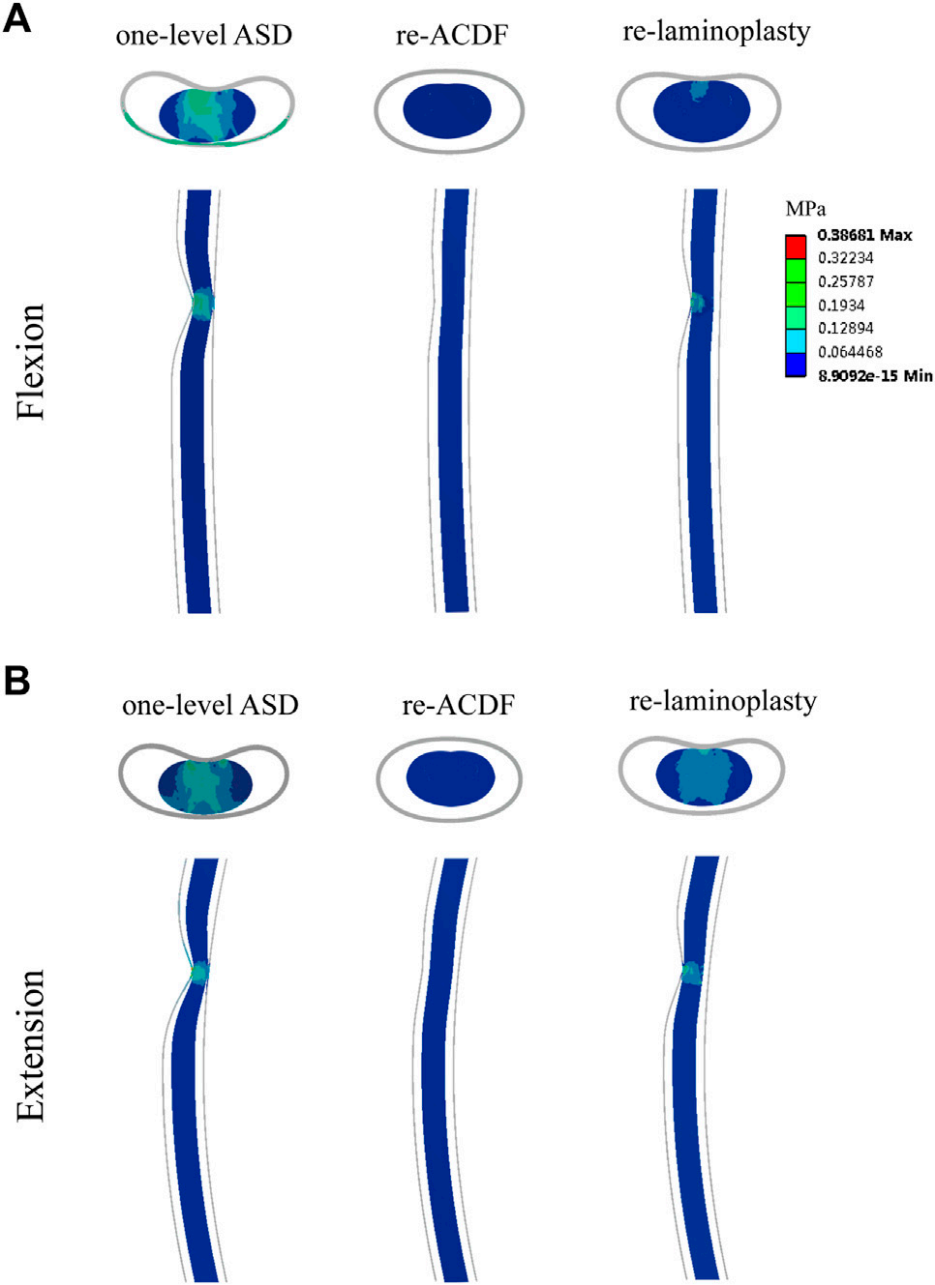
Distribution of von-Mises stress in the spinal cord of re-ACDF and re-laminoplasty models for the treatment of one-level ASD. **(A)** Stress distribution in flexion. **(B)** Stress distribution in extension.

**FIGURE 9:**
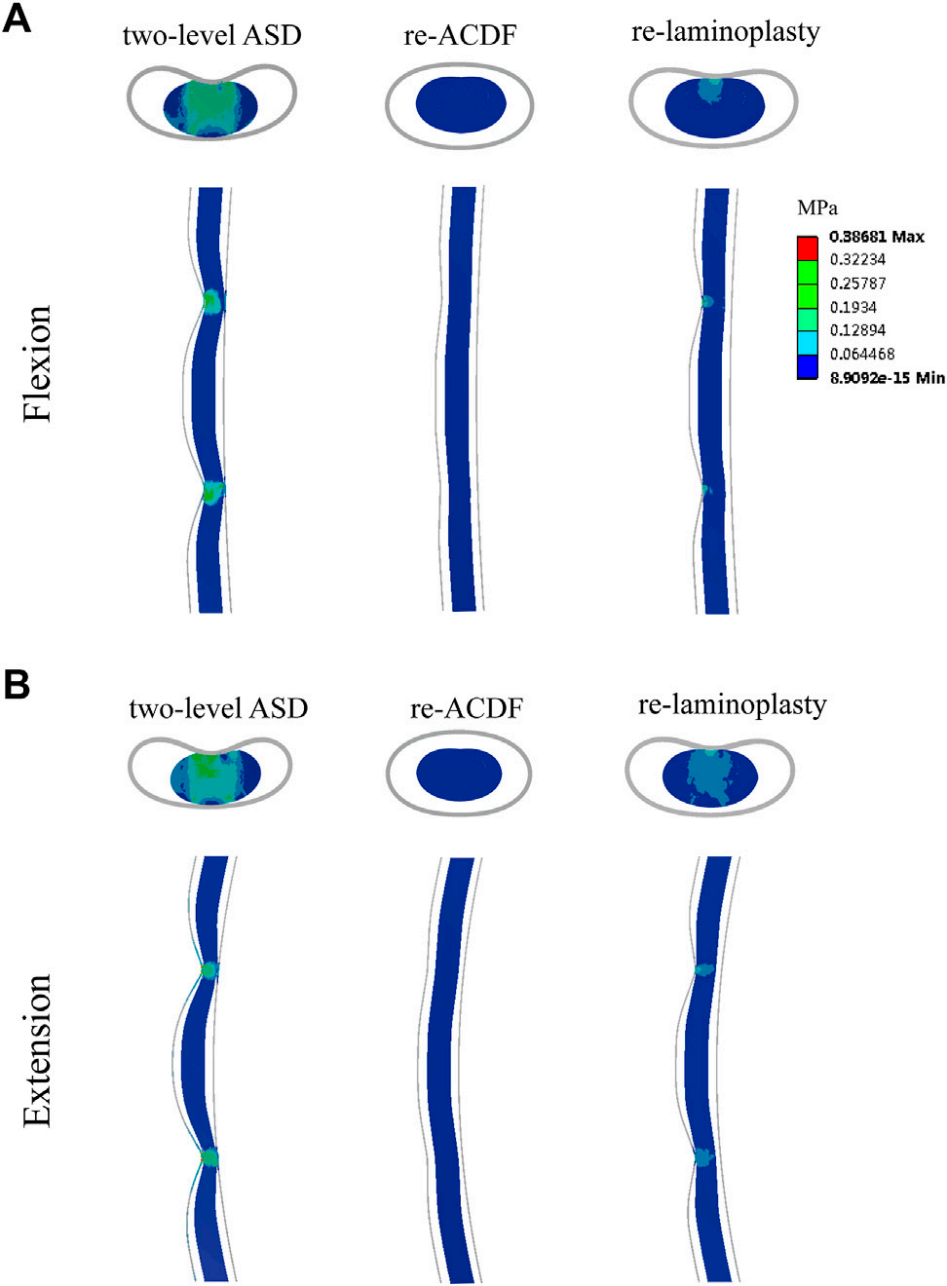
Distribution of von-Mises stress in the spinal cord of re-ACDF and re-laminoplasty models for the treatment of two-level ASD. **(A)** Stress distribution in flexion. **(B)** Stress distribution in extension.

The maximum von-Mises stress in the spinal cord of the FE models of different surgical approaches for the treatment of one-level or two-level ASD after primary ACDF were shown in Figure 7. The maximum von-Mises stress in the cord of the re-ACDF model was greatly reduced compared to the one-level or two-level ASD model. The stress in the cord of the re-laminoplasty model decreased to some extent, although it was higher than that of the re-ACDF model. The stress distribution in the spinal cord in the sagittal plane of different surgical approaches for the treatment of one-level or two-level ASD after primary ACDF were shown in Figures 8, 9. The peak stress occurred where the cord and dural sheath attached. Anterior and posterior surgical approaches all decreased the stress in the spinal cord caused by ASD after the first surgery.

## Discussion

The purpose of this study was to compare the biomechanical effects of second ACDF and laminoplasty for the treatment of one-level or two-level ASD after primary ACDF. The biomechanical results indicated that both ACDF and laminoplasty can relieve the increased stress in the spinal cord caused by ASD after primary ACDF, whereas ACDF can achieve a better decompression effect than laminoplasty regardless of the level of ASD. Revision surgery of the superior ACDF or the superior and inferior ACDF after primary ACDF both increased the ROM and IDP at the adjacent segments. Furthermore, laminoplasty after primary ACDF had no significant effect on the biomechanical stability of the spine.

Recently, symptomatic ASD has become a major problem after spinal fusion surgeries Some experts think that ASD is the result of a natural history, while others believe that ASD is due to compensatory pressure on adjacent discs following vertebral fusion (Hilibrand and Robbins, 2004; Kavadi and Badve, 2019). If ASD occurs, conservative treatment is often the first choice for many patients. However, a revision surgery should be considered for the patients with obvious clinical manifestation and poor effect of conservative treatment. There remains some debate on the appropriate surgical approaches for the treatment of ASD after ACDF. Revision surgery via anterior approach was reported to be effective for patients who underwent primary ACDF for symptomatic ASD (Li et al., 2016; O'Neill et al., 2016). However, the incidence of radiculopathy and ASD recurrence after anterior revision surgery was higher than that undergoing posterior approached (Xu et al., 2014). Furthermore, posterior revision surgery could result in greater blood loss and a longer hospital stay (Steinhaus et al., 2020). When patients developed spinal stenosis at the initial surgical levels or ossification of the posterior longitudinal ligament, a revision surgery with an anterior approach cannot easily resolve the issue, but a posterior approach can achieve extensive decompression (Cabraja et al., 2010; Cole et al., 2015).

ACDF and laminoplasty were reported to be effective in treating ASD after primary ACDF (Wang and Green, 2003; Basques et al., 2017). The clinical outcomes of the two surgical approaches have also been compared. The ACDF was reported to reduce intraoperative bleeding and better preserve cervical lordosis, while laminoplasty retained more ROM (Montano et al., 2019). Recently, Mohamed et al. reported a higher incidence of dysphagia, new-onset cervicalgia, and increased incidence of recurrence in patients with ACDF compared to those with laminoplasty (Mesregah et al., 2021). In a prospective cohort study of 60 patients with lordotic cervical spine, Liang et al. reported similar sagittal alignment results between ACDF and laminoplasty, while ACDF was associated with poor cervical lordosis preservation (Liang et al., 2019). Another study reported that both ACDF and laminoplasty can achieve favorable clinical results in patients with multilevel cervical spondylotic myelopathy (Chen et al., 2019). Compared with laminoplasty, ACDF has the advantage of less trauma and may be more suitable for elderly patients with poor surgical tolerance. However, the biomechanical evaluation of ACDF and laminoplasty for the treatment of ASD after ACDF is limited.

In the present study, the FE models of the one-level and two-level ASD based on C4-C6 ACDF were constructed to simulate the postoperative degeneration after primary ACDF. Revision surgeries of ACDF and laminoplasty were stimulated to compare the biomechanical effect of different surgical approaches for the treatment of ASD after primary ACDF. The biomechanical results suggested that revision surgery of the superior ACDF or the superior and inferior ACDF after the primary ACDF both increased the ROM and IDP at the adjacent segments. Increased IDP at the adjacent segments of the fused surgeries was supposed to be an important factor in the development of ASD (Eck et al., 2002). Xu et al. (2014) reported that patients who underwent a second ACDF after primary ACDF had a higher chance of developing recurrent ASD, up to 25%. The increased ROM and IDP at the adjacent segments of the re-ACDF model in our study may be a possible reason for the high incidence of recurrent ASD after second ACDF. Furthermore, no significant changes in the ROM and IDP of the re-laminoplasty model were observed, which is similar to the finding of a previous study (Xu et al., 2014). Decompression of the spinal cord is the main objective of revision surgery and it determines the outcome. The biomechanical results indicated that both ACDF and laminoplasty can decrease the stress in the spinal cord caused by ASD after primary ACDF, but ACDF can achieve a better decompression effect than laminoplasty regardless of the level of ASD. Compared with ACDF, laminoplasty serves as a motion-preserving procedure that allows for indirect decompression, which may be safer than direct decompression (Bakhsheshian et al., 2017).

Taken together, our results suggested that both ACDF and laminoplasty were effective for the treatment of ASD after primary ACDF. Although ACDF can achieve a better decompression effect, laminoplasty retained more ROM of the surgical segments. For decompression of one-level ASD after primary ACDF, both ACDF and laminoplasty were feasible and open to consideration. As for the superior and inferior ASD, multilevel laminoplasty may be a suitable choice, while ACDF could significantly increase the ROM and IDP at the adjacent segments. The biomechanical results of this study provided guidance for surgical decisions for the treatment ASD after primary ACDF, but the actual situation should also be considered in clinical practice.

There are some limitations in the present study. First, only linear elastic materials were used for the vertebral body and IVD, which ignored the anisotropic properties of materials. Second, the muscles and collagen fibers were not considered in this study, which may affect the stability of cervical spine. Third, the ligaments were considered as nonlinear Spring element with no effect on compression. Furthermore, the model was constructed based on the data from a single volunteer. Although it had the advantage of making comparisons between different conditions and treatments, the results were somewhat haphazard. However, the simplified model can objectively reflect the biomechanics of the spine and has certain clinical guiding value for the evaluation of different surgical methods. Meanwhile, more accurate FE model and clinical studies are needed to explore the effect of different surgical methods in the future.

## Conclusion

In conclusion, ACDF and laminoplasty can relieve the high level of stress in the spinal cord caused by ASD after primary ACDF, whereas ACDF can achieve a better decompression effect than laminoplasty regardless of the level of ASD. Revision surgery of the superior ACDF or the superior and inferior ACDF after primary ACDF increased the ROM and IDP at the adjacent segments, which may be the reason for the high incidence of recurrent ASD after second ACDF. Due to some defects in finite element analysis, it may not fully represent the real situation *in vivo*. The biomechanical results of this study provided guidance for surgical decisions for the treatment ASD after primary ACDF, but the actual situation should also be considered in clinical practice.

## Data Availability

The original contributions presented in the study are included in the article/Supplementary Material, further inquiries can be directed to the corresponding author.

## Supplementary Material

Supplementary Figure S1 Validation of IDP of the intact cervical model.
