# Supplementary figures and images for: Biomechanical Evaluation of Different Surgical Approaches for the Treatment of Adjacent Segment Diseases After Primary Anterior Cervical Discectomy and Fusion: A Finite Element Analysis
Source: Front Bioeng Biotechnol. 2021 Aug 31;9:718996. doi: 10.3389/fbioe.2021.718996 (PMC8438200; doi:10.3389/fbioe.2021.718996)

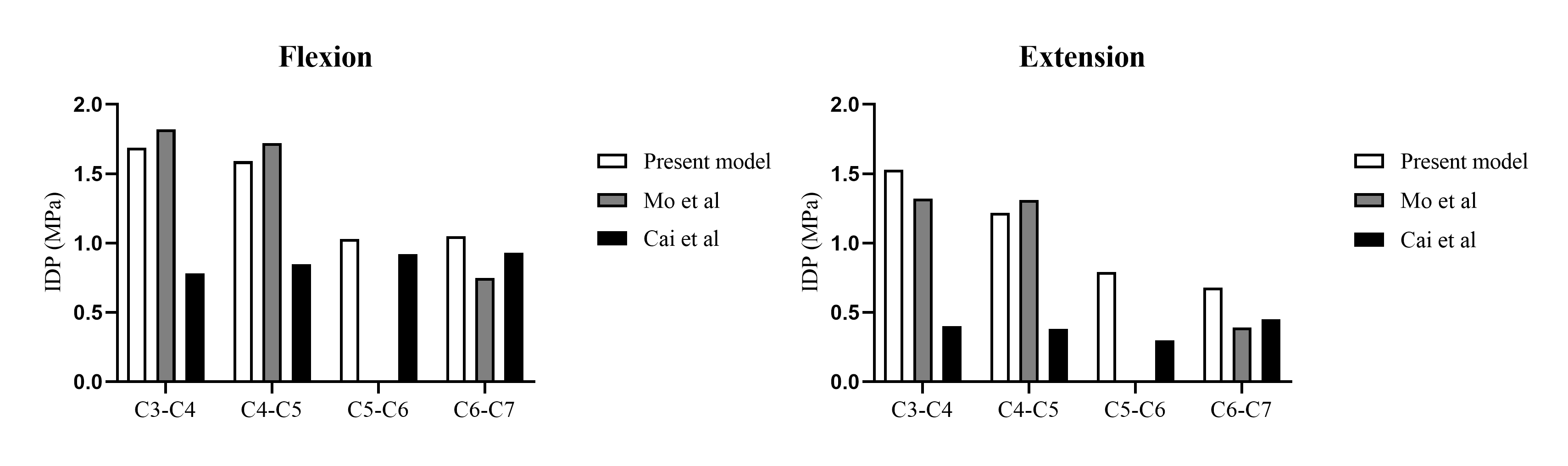

Supplement: Supplementary file 1 [file Image1.tif]
